# Salivary Calculus

**Published:** 1874-11

**Authors:** 


*Salivary Calculus, Etc.*—We are indebted to Dr. M. A. Berry, of Hagerstown, Maryland, for several very large specimens of Salivary Calculus, in one of which is imbedded the half of the root of an inferior central incisor. How the patient could tolerate such enormous deposits of this substance is a mystery.

These specimens have been placed in the Museum of the Baltimore College of Dental Surgery.

We are also indebted to Dr. J. D. Patterson, of Leavenworth, Kansas, for a plaster model of a mouth where almost half of the hard palate and alveolar process have been destroved by tertiary syphilis.

Also to Dr. J. W. Meng, of Lexington, Missouri, for a plaster model showing the effects of the inferior teeth on the alveolar process of the superior maxillary, after all of the upper teeth had been removed and no artificial ones substituted. The inferior teeth have made an indentation in the gum and alveolar process fully one half of an inch in depth.

Also to Dr. Jas. L. Nehon, of Lewisburg, West Virginia, for several teeth with exostosed roots, the removal of which relieved severe neuralgia.

All of these specimens have been placed in the Museum of the Baltimore College of Dental Surgery.

